# A Themed Issue in Honor of Professor K. Hüsnü Can Baser—Outstanding Contributions in the Fields of Pharmacognosy, Phytochemistry, Botany and Ethnopharmacology

**DOI:** 10.3390/molecules26185507

**Published:** 2021-09-10

**Authors:** İhsan Çalış, Györgyi Horváth, Agnieszka Ludwiczuk

**Affiliations:** 1 Department of Pharmacognosy, Faculty of Pharmacy, Near East University, Lefkoşa 99138, Cyprus; 2 Department of Pharmacognosy, Faculty of Pharmacy, University of Pécs, 7624 Pécs, Hungary; horvath.gyorgyi@gytk.pte.hu; 3 Independent Laboratory of Natural Products Chemistry, Department of Pharmacognosy, Faculty of Pharmacy, Medical University of Lublin, 20-093 Lublin, Poland; aludwiczuk@pharmacognosy.org

Dear Colleagues,

This Special Issue is in honor of Prof. Dr. K. Hüsnü Can Başer and his outstanding contributions in the fields of pharmacognosy, phytochemistry, botany, and ethnopharmacology. His major areas of research include essential oils, alkaloids, and biological, chemical, pharmacological, technological, and biological activity research into natural products. Prof. Başer, a pharmacist, obtained his Ph.D. in pharmacognosy from the University of London (1978). He is the author of 1257 scientific contributions, including 861 papers in international peer-reviewed journals, 193 papers in Turkish journals, 142 papers in conference proceedings, 61 books and book chapters, as well as 19 project reports. He communicated 1040 papers at 304 symposia and conferences. According to Science Citation Index (SCI) his 611 articles have been cited 12,165 times, corresponding to an h-index of 50. According to Google Scholar, his articles have been cited 32,089 times, which corresponds to an h-index of 77. His i10 index is 604.

He has taken part in and implemented numerous national and international development and research projects. He served as Dean of the Faculty of Pharmacy at Anadolu University (1993–2001), Vice Dean of the Faculty of Pharmacy (1982–1993), Head of the Department of Professional Pharmaceutical Sciences (1982–1993), Head of the Pharmacognosy Section (1982–ongoing), Member of the University Board and Senate (1982–2001; 2007–2010 (Senate)), and Director of the Medicinal and Aromatic Plant and Drug Research Centre (TBAM) (1980–2002) in Anadolu University. Between 1984 and 1994, he was appointed as the National Project Coordinator of Phase I and Phase II of the UNDP/UNIDO projects of the Government of Turkey titled “Production of Pharmaceutical Materials from Medicinal and Aromatic Plants”, through which TBAM was strengthened. Between 1988 and 1997, he organized 10 in-plant group training programs for the benefit of scientists in developing countries. The program titled “Training in the Utilization of Medicinal and Aromatic Plants in Pharmaceutical and Related Industries (TRUMAP)” was held every September for 25 days for 10 selected participants from among those nominated by developing countries. The program was co-financed by the Government of Turkey and United Nations Industrial Development Organization (UNIDO) and the only of its kind in the world. One hundred participants from 40 countries were trained in 10 programs. He also worked as a UNIDO Consultant in Nigeria, Ghana, Sierra Leone, Sudan, and Iran for a total of 6 months to develop projects on the industrial utilization of indigenous medicinal and aromatic plants. Between October 6 and 26, 1997, he worked as a UNIDO consultant on the quality improvement of essential oils in South Africa, Zambia, Malawi, Uganda, Ethiopia, and Ghana and worked as a short-term consultant on a UNIDO project on essential oils in November–December 2012 in Egypt; at an FAO project on medicinal and aromatic non-wood forest products in February 2013 in Uzbekistan; and at a UNIDO project on fragrances in August 2018 in Oman. He worked as a national point of contact for the Asian-Pacific Information Network of Medicinal and Aromatic Plants (APINMAP) (1992–1997) and Useful Plants of the Mediterranean Network (MEDUSA) in Turkey (1996–2000). He served as Member of the Turkish Pharmacopoeia Commission (1985–2012), Member of the Registration Commission for Plant Drugs at the Ministry of Health (1985–1992), Member of the No. 11 Group of Experts of the European Pharmacopoeia Commission (1995–1999), Member of the 13B Group of Experts of the EP Commission (1999–2012), and as a Member of the TCM Working Party of the EP Commission (2008–2012).

He served as Secretary-General of the International Council of Medicinal and Aromatic Plants (ICMAP) between November 1997 and February 2003. In February 2003 in Chiang Mai (Thailand), he was elected as the Vice President of ICMAP. In November 2008 in Cape Town, South Africa, he was elected as the President of ICMAP. In August 2014 in Brisbane, Australia, he was elected as Vice President responsible for organizing WOCMAP VI in 2019 in Famagusta, North Cyprus. He is currently serving as a member of the ICMAP Bureau. He was appointed as a member of the Executive Committee of the Academy of Pharmacy of the Turkish Pharmacists’ Association in 2006, and as its President in February 2008, a position he served in until 2012. As a result of agreements established between the Institute of the Chemistry of Plant Substances (ICPS) of the Uzbekistan Academy of Sciences and the Medicinal and Aromatic Plant and Drug Research Centre (TBAM) of Anadolu University in 1993, exchanges of many research scientists were established between the two institutions and from 1994 onwards a biennial scientific symposium titled “International Symposium on the Chemistry of Natural Compounds (SCNC)” has been organized alternately in Uzbekistan and Turkey (1st 1994 Tashkent; 2nd 1996 Eskisehir; 3rd 1998 Bukhara; 4th 2001 Isparta; 5th 2003 Tashkent; 6th 2005 Ankara; 7th 2007 Tashkent; 8th 2009 Eskisehir; 9th 2011 Urumqi (China); 10th 2013 Tashkent; 11th 2015 Antalya; 12th 2017 Tashkent; 13th 2019 Shanghai;14th 2021 Tashkent). He is the founder of the Herbarium of the Faculty of Pharmacy at Anadolu University (Acronym = ESSE), founding member and President (until 2002) of the Turkish Society of Cosmetic Scientists (TCoS). He was a founding member of the Turkish Phytotherapy Society and acted as founding member and Vice President of the Society for Flora Research founded in April 2005 in Istanbul, Turkey. He has organized over 50 national and international scientific meetings since 1987. He is a member of the World Health Organization (WHO) Expert Advisory Panel on Traditional Medicine (2007–2015). His term was recently extended. He was an elected member of the Advisory Board of GA (The Society for Medicinal Plant and Natural Product Research) (2009–2014) and organized the 59th International Symposium of GA in September 2011 in Antalya, Turkey.

Since February 1998, he has been a Fellow of the Linnean Society (FLS), the most prestigious society of biological sciences based in London. He is the recipient of many national and international awards: 1995 Recipient of the Distinguished Service Medal of IFEAT (International Federation of Essential Oils and Aroma Trades) based in London, UK, “Memorial Silver Medal” of the Scientific Partnership Foundation of Russia for promoting scientific partnership among young scientists and for his contribution to the development of science (Tashkent, 2003), “Academician Award” (Istanbul, 2004), “Centennial Success in Career Award” of Rotary Turkey (Hatay, 2005), first recipient of the “Science Award” of the Academy of Pharmacy of the Turkish Pharmacists’ Association (Ankara, 2005), “Science Award” (Health Sciences) of the Scientific and Technological Research Council of Turkey (TUBITAK) (Ankara, 2005), “Science Award” of the Society of Contemporary Journalists Eskisehir (Eskisehir, 2006), Recipient of 2008 “Science Award” (Health Sciences) of Popular Science Magazine (Ankara, 2009), ISHS (International Society for Horticultural Science) Medal, (Brisbane, Australia 2014), votive tablet of the Academy of Pharmacy of the Turkish Pharmacists Association (Ankara, 2017), Publication Award of Near East University in 2017, 2018, and 2019 and Publication Honor Award in 2018, the Best Academician Award of Near East University in 2019, Altin Havan (Golden Mortar) Service Award of *Eczaci* journal (Istanbul, 2019), Publication Award of Near East University in 2020. His name appeared 35th in the ranking of the *100 Turks Guiding Science* published by Sanko Holding in May 2017. He ranked 22nd among the 950 Turkish scientists listed in the “Most Influential Scientists of the World” list in September 2020.

He is co-Editor of the book *Essential Oils: Science, Technology and Applications* together with Prof. Dr. Gerhard Buchbauer of Vienna University, CRC Press (Taylor & Francis), January 2010. A second edition was published in November 2016 and won the James A. Duke Excellence in Botanical Literature Award of the American Botanical Council in March 2017. The third edition of the book appeared in August 2020. The memorial book *Essential Oils* by N. Kirimer and A. Mat in 1999 was published in honor of Prof. Dr. K. Hüsnü Can Başer on his 50th birthday. The *Natural Product Communications* journal dedicated a Special Issue (5(9), 2010) in honor of Prof. Başer’s 60th birthday (September 2010). A memorial Special Issue for his 70th birthday was published in 2021 in the same journal. He retired from his job Anadolu University on 16 February 2011, and worked as a Visiting Professor at the Botany and Microbiology Department of the College of Science of King Saud University in Riyadh, Saudi Arabia, giving lectures and implementing research projects. He was appointed as an International Board Member of the Organization for the Phyto Taxonomic Investigation of the Mediterranean Area (OPTIMA) (2013–2019). He worked for a short time as Director of the Technology Transfer Office (TTO) at Bahcesehir University in Istanbul, Turkey (November 2013–July 2014). He was elected as Member of the Science Academy of Turkey on 27 November 2016. Prof. Başer is among the group of scientists who named five new plant species and identified two plant species as new record for the flora of Turkey. Four plants have been named after him in recent years: *Origanum husnucan-baseri* H.Duman, Z.Aytac et A.Duran and *Aristolochia baseri* Malyer et Erken, *Centaurea baseri* Kose et Alan, *Thymus baseri* Öztürk, Yaylacı, Koyuncu et Ocak. He is the joint Editor of the second supplement (Volume 11) to the *Flora of Turkey and the East Aegean Islands* together with the late Prof. P.H. Davis (2000). Prof. Başer and Prof. F. Demirci founded a biotechnology company (BadeBIO) at the Eskisehir Technopark (ATAP) (November 2009). Since 2016, he has been working as Head of the Pharmacognosy Department at the Faculty of Pharmacy, Director of the Graduate Institute of Health Sciences, and finally Director of the Institute of Graduate Studies, and member of the Executive Board and the Senate at the Near East University in Nicosia (Lefkoşa), Northern Cyprus.

This Special Issue in honor of Prof. Baser consists of twelve articles in the form of eight research articles and four reviews. Our goal, as Guest Editors, was to make a gesture acknowledging the tremendous impact of Prof. Baser’s work on the fields of pharmacognosy, phytochemistry, botany, and ethnopharmacology. We are very pleased to see the completion of this Special Issue that contains contributions from many of his colleagues and collaborators.

Most of the research articles included in this issue are focused mainly on the isolation of compounds occurring in plants. One paper, prepared by Iklas A. Khan group [1], described the isolation of one new and seven known sesquiterpenoids from copaiba oil. The new compound was elucidated as (*E*)-2,6,10-trimethyldodec-8-en-2-ol by use of 1D and 2D NMR and GC/Q-ToF mass spectral data analyses. The authors concluded that the isolated constituents could be used as chemical markers to evaluate the safety or quality of copaiba oil, which has been used in folk medicine for centuries as a wound-healing agent. The studies on two *Aloe* species, *A. vera* and *A. plicatilis*, by Rauwald et al. Another paper, [2], led to the isolation of series of phenolic polyketides such as naphthalenes (hexaketides), chromones (heptaketides), anthrones/anthraquinones (octaketides), and nonaketides. For this, Sephadex-LH20 gel filtration, combined silica gel 60- and RP18-column chromatography, as well as high-speed counter-current chromatography (HSCCC) were used. The structures of all polyketides were elucidated by ESI–MS and 2D 1H/13C NMR (HMQC, HMBC) and CD spectroscopy. The isolated metabolites were further screened in vitro to identify *Aloe* polyketides with anti-inflammatory potential.

Bioguided isolation of volatile components with repellent activity from the essential oils obtained from *Pinus* and *Juniperus* species was carried out by Leandros A. Skaltsounis group [3]. To perform this work, they use centrifugal partition chromatography (CPC), a liquid–liquid separation method, as an isolation technique, gas chromatography–mass spectrometry (GC/MS) as an analytical tool to monitor the isolation process and, finally, all isolated compounds, fractions, as well as essential oils were evaluated for their repellent properties against *Aedes albopictus*. The essential oils from needles of *Pinus pinea* and fruits of *Juniperus oxycedrus* subsp. *deltoides* turned out to be the most active against *Ae. albopictus*, and of these isolated oil compounds, (−)-limonene, guaiol, germacrene D, and mixtures of (−)-limonene/β-phellandrene presented significant repellent activity (>97% repellency). This methodology could be a valuable tool in the effort to develop potent mosquito repellents which are environmentally friendly.

Centrifugal partition chromatography (CPC) in combination with normal phase flash chromatography and reversed phase preparative HPLC was used in the isolation of sesquiterpenes from myrrh resin by Jörg Heilmann and coworkers [4]. From the ethanolic extract, sixteen compounds were isolated. Two of them, 9-nor-9,10-seco-isolindestrenolide and 9,10-seco-isohydroxylindestrenolide, are novel 9,10-seco-eudesmanes and exhibited an unprecedented sesquiterpene carbon skeleton. The isolated compounds were tested in an in vitro ICAM-1 assay, and only lindestrenolide acted as moderate inhibitor of this adhesion molecule ICAM-1 with an IC_50_ value of 44.8 μM.

Shaza M. Al-Massarani et al. [5] reported on the isolation of five phenolic compounds from the aerial parts of the Saudi plant *Nuxia oppositifolia*. These were two flavones, hispidulin and jaceosidin, and three phenylethanoid glycosides, verbascoside, isoverbascoside, and conandroside. The isolated flavones together with eleven previously isolated compounds were evaluated against the Yellow Fever mosquito, *Aedes aegypti*. Four compounds (β-sitosterol and oleananne-type triterpenoids) displayed adulticidal activity with LD_50_ values of 2–2.3 μg/mosquito. All isolated compounds exhibited notable free radical scavenging properties, compared with the positive control.

In their research article, Györgyi Horváth and colleagues [6] focused on the isolation and identification of carotenoids from the aerial parts of *Melilotus officinalis* and on the effect of isolated (all-*E*)-lutein 5,6-epoxide on primary sensory neurons and macrophages involved in nociception as well as neurogenic and non-neurogenic inflammatory processes. The main carotenoid was isolated by column liquid chromatography (CLC), identified by spectroscopic methods, and the isolation process was monitored by HPLC technique. Then, the isolated (all-*E*)-lutein 5,6-epoxide was packed into RAMEB (randomly methylated-β-cyclodextrin) to enhance its water solubility for in vitro functional experiments. The results showed that the tested compound suppressed mustard oil (MO)-induced TRPA1 receptor activation on primary sensory neurons, which opens perspectives for its analgesic and anti-inflammatory application.

Microbial biotransformation is an important tool in drug discovery and for metabolism studies. Erdal Bedir et al. [7] reported on the products formed during the biotransformation of a cardenolide, gitoxigenin, by the endophytic fungus *Alternaria eureka* 1E1BL1. Gitoxigenin was obtained, by the authors, from oleandrin isolated from the leaves of *Nerium oleander*, which was subjected to an acid-catalyzed hydrolysis. After 21 days of the incubation process, eight compounds were isolated, five of which were new cardenolides. Structural elucidations were accomplished through 1D/2D NMR, HR-ESI–MS and FT-IR analysis. The isolated compounds were tested for their cytotoxic activity, and 3-epi-diginatigenin exhibited the highest activity against A549, PANC-1, and MIA PaCa-2 cells without causing toxicity on healthy cell lines (MRC-5 and HEK-293).

Cholinesterase inhibition is an important treatment strategy for Alzheimer’s disease, as acetylcholinesterase (AChE) and butyrylcholinesterase (BChE) are involved in the pathology of this disease. Ilkay Erdoğan Orhan et al. [8] reported the cholinesterase inhibitory potential of 24 natural products from different chemical classes. Among them, only a steroidal sapogenin, smilagenin, and an alkaloid, kokusaginine, displayed inhibitory action against AChE. BChE was inhibited by only methyl rosmarinate and kokusaginine. Molecular docking experiments showed that the orientation of smilagenin and kokusaginine was mainly driven by the interactions with the peripheral anionic site (PAS) comprising residues of hAChE, while kokusaginine and methyl rosmarinate were able to more deeply access the active gorge in hBChE. These data indicate that similagenin, kokusaginine, and methyl rosmarinate are promising compounds for designing novel anti-Alzheimer’s agents.

Four review papers complete this Special Issue. The first, written by Ryan D. Rattray and Ben-Erik Van Wyk [9], provides comprehensive insight into the Lamiaceae flora of southern Africa, comprising 297 species in 42 genera, 105 of which are endemic to the subcontinent. The main aim of their study was to present an up-to-date account of the botany, chemistry, and traditional uses of the family in southern Africa. In another review paper by Andrea Vasas and colleagues [10], the information concerning stilbenoids occurring in Cyperaceae species is summarized. The authors focused on the isolation, synthesis, and pharmacological properties of this group of compounds in addition to their chemotaxonomical significance. The chemistry of the cannabinoids and major non-cannabinoid constituents (terpenes, non-cannabinoid phenolics, and alkaloids) occurring in *Cannabis sativa*, with special emphasis on their chemical structures, methods of isolation, and identification, is the subject of the next review prepared by Mohamed M. Radwan et al. [11] that was included in this Special Issue. Sansei Nishibe and coworkers [12] summarized their research studies on the biological effects of forsythia (*Forsythia suspensa*) leaves containing phillyrin and other polyphenolic compounds, particularly against obesity, atopic dermatitis, and influenza A virus infection, and its potential as a phytoestrogen. The authors also conclude that forsythia leaves are useful and safe as a health food containing a PDE4 inhibitor, supporting its use in the treatments of metabolic disorders and inflammatory dysregulation.

Overall, the 12 manuscripts published in the current SI cover many aspects of research conducted in the fields pharmacognosy, phytochemistry, botany, and ethnopharmacology and, we hope, provide interesting new information for scientists working in these fields.

## Data Availability

Not applicable.
